# Non-Alcoholic Fatty Liver Disease: Metabolic, Genetic, Epigenetic and Environmental Risk Factors

**DOI:** 10.3390/ijerph18105227

**Published:** 2021-05-14

**Authors:** Oriol Juanola, Sebastián Martínez-López, Rubén Francés, Isabel Gómez-Hurtado

**Affiliations:** 1 Gastroenterology and Hepatology, Translational Research Laboratory, Ente Ospedaliero Cantonale, Università della Svizzera Italiana, 6900 Lugano, Switzerland; oriol.juanola.juarez@usi.ch; 2 Clinical Medicine Department, Miguel Hernández University, 03550 San Juan de Alicante, Spain; sebastian.martinez@goumh.umh.es (S.M.-L.); frances_rub@umh.es (R.F.); 3 Alicante Institute for Health and Biomedical Research (ISABIAL), Hospital General Universitario de Alicante, 03010 Alicante, Spain; 4 Networked Biomedical Research Center for Hepatic and Digestive Diseases (CIBERehd), Institute of Health Carlos III, 28029 Madrid, Spain

**Keywords:** non-alcoholic fatty liver disease, non-alcoholic steatohepatitis, obesity, metabolic syndrome, type 2 diabetes mellitus

## Abstract

Non-alcoholic fatty liver disease (NAFLD) is one of the most frequent causes of chronic liver disease in the Western world, probably due to the growing prevalence of obesity, metabolic diseases, and exposure to some environmental agents. In certain patients, simple hepatic steatosis can progress to non-alcoholic steatohepatitis (NASH), which can sometimes lead to liver cirrhosis and its complications including hepatocellular carcinoma. Understanding the mechanisms that cause the progression of NAFLD to NASH is crucial to be able to control the advancement of the disease. The main hypothesis considers that it is due to multiple factors that act together on genetically predisposed subjects to suffer from NAFLD including insulin resistance, nutritional factors, gut microbiota, and genetic and epigenetic factors. In this article, we will discuss the epidemiology of NAFLD, and we overview several topics that influence the development of the disease from simple steatosis to liver cirrhosis and its possible complications.

## 1. Introduction

NAFLD has become, in recent years, one of the most common liver diseases in the world. It is based on different liver disorders characterized by accumulation of fat (hepatic steatosis) in more than 5% of hepatocytes [1], primarily in the form of triacylglycerols, due to an alteration of the homeostatic mechanisms that regulate synthesis of fat in the liver [2,3,4]. These changes occur without other causes of secondary fat accumulation in the liver such as excessive alcohol consumption (<20 g ethanol/day), viral or autoimmune hepatitis, congenital hepatic disorders, or long-term use of steatosis-inducing medications [5]. However, NAFLD is often presented together with type 2 diabetes mellitus (T2DM), obesity, dyslipidemia, and hypertension, thus constituting a cardiometabolic disease [6].

The term NAFLD has undergone an evolution throughout history, as knowledge about the disease and diagnostic methods have advanced [7]. Recently, experts in fatty liver disease have agreed that the term NAFLD does not reflect the knowledge that currently exists about the metabolic dysfunction associated with the disease. “MAFLD”, metabolic associated fatty liver disease, has been suggested as a more proper overarching term. MAFLD, as the earlier NAFLD, represents the hepatic manifestation of a multisystem disorder, which is heterogeneous in its underlying causes, presentation, course, and outcomes [8]. In this review, we will use the term NAFLD because it is the expression used in most of the articles included and reviewed for our work.

NAFLD can have different forms of presenting itself, from simple accumulation of fat that is a metabolic disorder that does not present symptoms (non-alcoholic fatty liver (NAFL)) to a symptomatic non-alcoholic steatohepatitis (NASH). NAFLD is asymptomatic in most cases and is associated with obesity and characteristics of metabolic syndrome (MS), as mentioned before [9,10]. NASH, on the other hand, is characterized by steatosis, hepatocellular ballooning, lobular inflammation, and often fibrosis [11]. In an effort to regenerate the damaged tissue, hepatocytes are replaced by type I collagen produced by stellate cells, leading to the progression of NASH toward [12,13] fibrosis and cirrhosis with their overt clinical consequences [14].

The prevalence of NAFLD evolves in parallel with obesity and varies among countries and ethnicities. Globally, it has been estimated at around 25% in the general population [6], increasing up to 55.5% in patients with T2DM [14]. Most of the studies on NAFLD epidemiology are performed in the USA where the prevalence has been established in 24.13% [6], being the lowest among African Americans, followed by European Americans, and then Hispanic Americans [15]. These results are supported by lower NAFLD prevalence rates observed in the general population of Africa (13.48%) while higher ones have been observed in South America and Middle East (30.45% and 31.79%, respectively) [6]. The prevalence trend observed in Europeans is calculated at 23.71% [6], similar to the USA, and there is evidence that points toward an increasing gradient from the south to the north of Europe [15]. In Asia, the epidemiology of NAFLD is similar to Western countries with a prevalence estimated in 25% where urban or rural origin and lean NAFLD—a topic treated in the obesity section—play major roles [16,17]. Despite the literature concerning NAFLD being abundant as it can be diagnosed by non-invasive methods, the precise prevalence of NASH in the general population remains unknown as it requires confirmatory liver biopsy. Indirect estimations using data from the American population have calculated the prevalence of NASH in the general population in a 3–5% range [6,15,18]. Therefore, only a small proportion of subjects with NAFLD develop NASH with a consequent higher risk of complications such as liver fibrosis, cirrhosis, and hepatocellular carcinoma (HCC). While patients with NAFL have similar life expectancy than the general population, NASH patients have a lower survival, mainly due to cardiovascular causes and the progression of liver damage.

The number of patients in the general population with NAFLD who develop NASH is unknown, but it is greater than 10% of the overall NAFLD population [19]. Primary studies situated the prevalence of biopsy-proved NASH among NAFLD patients in 29.9% [20], but during the last years, this rate has been augmented to 59.1% [6], representing the increasing burden of NAFLD and NASH worldwide. In line with this, a mathematical model suggests an increasing trend in the cases of advanced stages of liver disease and liver-related deaths among the NAFLD population in the upcoming years [21].

**Figure 1:**
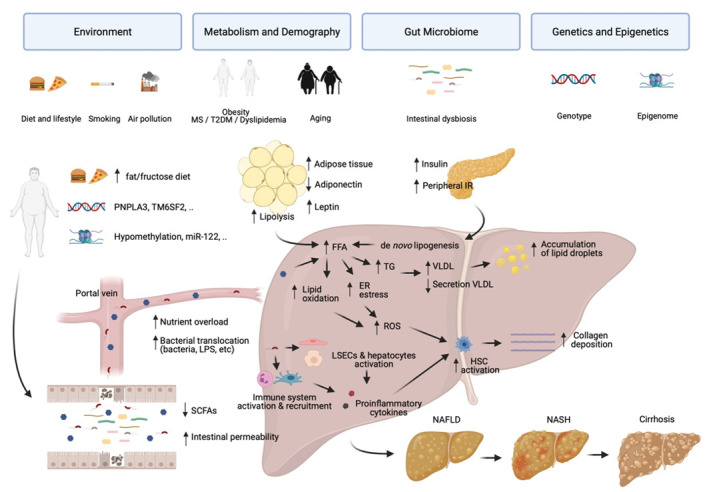
Major risk factors and pathophysiology of NAFLD. Genetically susceptible individuals under adverse environmental conditions including smoking, air pollution, and/or poor diet enriched in fat/fructose are prone to developing NAFLD. Obesity, MS, T2DM, dyslipidemia, and age increase the risk for fatty liver disease. Increased energy uptake due to high fat diet leads to an increased body fat, peripheral tissue insulin resistance, and metabolic syndrome. Augmented lipolysis, de novo lipogenesis, and constant absorption of high-energy nutrients induce increased FFAs and ultimately hepatic steatosis. Lipotoxicity, due to increased levels of lipids in the liver, induce production of ROS due to increased lipid oxidation and ER stress. Poor diet in vulnerable patients may induce intestinal dysbiosis associated with reduced production of SCFAs and increased intestinal permeability. Bacterial translocation of bacteria or its products to the liver result in activation of immune cells, hepatocytes, and LSECs, and release of proinflammatory cytokines. Production of ROS and proinflammatory cytokines drive the activation of HSCs and deposition of collagen inducing fibrosis and progression of liver disease from simple steatosis to steatohepatitis, cirrhosis and hepatocellular carcinoma. MS: Metabolic syndrome; T2DM—Type 2 diabetes mellitus; SCFAs—Short-chain fatty acids; FFA—Free fatty acids; TG—Triglyceride; VLDL—Very low-density lipoprotein; ER—Endothelial reticulum; ROS—Reactive oxygen species; LSECs—Liver sinusoidal endothelial cells; HSC—Hepatic stellate cells; NAFLD—Non-alcoholic fatty liver disease; NASH—Non-alcoholic steatohepatitis. This figure was created using the BioRender platform.

Complex interactions among environmental factors, metabolism and demography, genetic variants, and gut microbiota are involved in the pathogenesis of NAFLD [15]. Current understanding of the disease involves excessive hepatic accumulation of lipids resulting from an increased fatty acid input/output balance because of changes in the lifestyle associated with high energy uptake, sedentarism, and MS. The augmented free fatty acid uptake in the liver results from high energy diet, increased lipolysis of adipose tissue, and de novo hepatic lipogenesis, while output mechanisms related with fatty acid oxidation and very low-density lipoproteins secretion (VLDL) remain insufficient to compensate for the accumulation of triglycerides [16]. Saturated fatty acids in the liver induce lipotoxicity associated with increased endoplasmic reticulum stress [17,18] and lipid oxidation responses that increase the oxidative stress in the liver, a topic analyzed in depth by Bovi et al. [19]. In addition, poor diet in patients with NAFLD may contribute to changes in the microbiota related with low production of short chain fatty acids (SCFAs), increased intestinal permeability, and translocation of luminal bacteria or its products to the liver where they will foster an inflammatory environment. Consequently, hepatic stellate cells will activate and release collagen, contributing to fibrosis and progression of the disease. The contribution of all these factors can be followed in detail in Figure 1 and, together with the genetic and epigenetic background, will be the topics addressed in this review.

## 2. Metabolic Risk Factors

Currently, different studies have shown a strong association between NAFLD and each of the risk factors associated with MS [20,21,22], especially obesity, T2DM, and dyslipidemia [5].

### 2.1. Obesity

Obesity is considered the main risk factor for NAFLD, since body mass index (BMI) and waist circumference correlate positively with both the presence of NAFLD and disease progression [23]. Interestingly, NAFLD patients between their 40s and 60s are likely to be obese. In fact, the entire range of obesity is associated with NAFLD, from being overweight to obese and severely obese. In this setting, more than 95% of severely obese patients undergoing bariatric surgery will develop NAFLD [24,25,26,27], highlighting the close pathogenic connection between these two disorders.

Nevertheless, a proportion of patients with NAFLD and normal body mass index (BMI) represent the already known lean or non-obese NAFLD. This condition lacks the complications associated with obesity, so it is expected to be less severe than in obese NAFLD [28], but data in this regard require further study. The lack of evident risk factors in this specific population results in an under-diagnosed liver disease with a global prevalence that ranges from 5–26% [29]. Lean NAFLD prevalence is particularly relevant in rural areas of Asia where the rates reach up to 30% compared to 7% in Americans [30]. In general, patients with lean NAFLD have an altered metabolism associated with higher circulating triglycerides and IR compared to healthy individuals, but lower waist circumference and prevalence of MS compared to obese NAFLD patients [31]. Even if lean NAFLD individuals can present moderate metabolic alterations, they are at risk of progressing to NASH and advanced fibrosis [32].

Lipotoxicity and glucotoxicity play a crucial role in the development of simple steatosis in the liver and its progression to NASH. In this regard, a high fat and carbohydrate diet, which obese patients are more prone to follow, favors fat deposition in the liver by different mechanisms including mitochondrial defects and endoplasmic reticulum and oxidative stress, as mentioned [15,33]. Then, if this stage of simple steatosis in the liver is not successfully managed, an intra-hepatic inflammatory process is triggered [34]. As a consequence, innate immune cells in the liver become activated and infiltrate the hepatic tissue, releasing cytokines that further promote inflammation, also contributing to the onset of the fibrogenic process that usually appears when inflammation is prolonged [35]. Obesity also causes the secretion of adipokines (e.g., leptin, adiponectin) and hormones in the liver, which can contribute to the progression of NAFLD to NASH, cirrhosis, and HCC [35,36].

### 2.2. Metabolic Syndrome

MS has multiple definitions, but some of its agreed manifestations are increased waist circumference, hyperglycemia, dyslipidemia, and systemic hypertension [37]. MS incidence, as in the case of NAFLD, has increased in recent years and both diseases are considered to be closely linked [21,38,39]. In fact, the association between NAFLD and MS features is typically bidirectional, especially with respect to diabetes and hypertension, since MS increases the risk of NAFLD, whereas NAFLD and NASH treatment could also improve some of the features of MS. This fact is important since MS is also a factor that promotes adverse cardiovascular events and increases mortality in patients with NAFLD [38,39].

In MS patients, the capacity of insulin to inhibit glucose production is reduced, resulting in mild hyperglycemia, which in turn stimulates insulin secretion, causing a state of hyperinsulinemia. Insulin normally decreases the amount of VLDL, by suppressing its hepatic production, or by inhibiting lipolysis of adipose tissue [40]. In patients with NAFLD or MS, insulin does not suppress either the production of triglyceride-rich VLDL particles from the liver or lipolysis [41], which is the main mechanism that contributes to the increase in serum triglycerides in both patients with MS and NAFLD [40]. This dyslipemia is characterized by a reduction in HDL cholesterol as well as the production of small, dense LDL particles that are highly atherogenic [42], due to the rising levels of VLDL. As a consequence, these patients have higher risks of developing cardiovascular disease.

### 2.3. Diabetes and Insulin Resistance

T2DM has a strong relationship with the progression of NAFLD, in fact more than 50% of patients with T2DM have NAFLD [43,44]. Diabetes is not only a common comorbidity of NAFLD, but also one of the determinants of the progression of NAFLD toward NASH, developing accelerated liver fibrosis and HCC [45,46,47,48,49]. As with MS, T2DM predisposes to NAFLD, and the reverse is also true.

IR, on the other hand, is considered one of the critical cellular abnormality that causes the development of both NAFLD and T2DM, and has been recognized as an integral component of NAFLD pathogenesis [50], worsening with disease progression. In the liver, IR is characterized by increased gluconeogenesis and decreased hepatic glycogen synthesis [51]. Insulin-stimulated liver glycogen synthesis, in turn, is negatively correlated with liver fat content [51]. As a consequence, NAFLD patients, as in the case of MS, also have a higher risk of incident diabetes [52].

It has been increasingly recognized that inflammatory pathways are critically involved in IR development. However, is still unclear when the inflammatory processes begin [53,54]. Moreover, the etiology of IR is complex and involves many other different pathways besides inflammation [55]. For instance, in addition to the existing dysregulation in the metabolism of fatty acids, gastrointestinal tract disbiosis could be one of the early events to occur in the evolution of both IR and NAFLD [56].

## 3. Gut Microbiome Composition

The human gut microbiome is made up of 10–100 trillion microorganisms, mainly bacteria, with this number about ten times higher than the number of eukaryotic human cells [57], susceptible to environmental and pathophysiological alterations [58]. Recent years have allowed a better understanding of the active role of intestinal microbiota in human physiology. The most common bacterial phyla are *Bacteroidetes* and *Firmicutes*, while the predominant prokaryotic microorganisms are *Euryarchaeota* [59]. The composition and function of the microbiota are determined by a variety of host and environmental factors including diet, geographic location, physical activity, and medication.

Recent studies have shown that the gut microbiota affects hepatic carbohydrate and lipid metabolisms and also influences the balance between pro-inflammatory and anti-inflammatory effectors in the liver, which directly affects NAFLD and its progression to NASH. Several experiments based on manipulation of the microbiome provide the most important evidence on the role of the gut microbiome in obesity and NAFLD in mice and humans. In an interesting study, germ-free mice fed with a high-fat diet exhibited lower levels of liver lipids compared to conventionally housed mice [60]. In another study, microbiome transferred from mice that developed fasting hyperglycemia and insulinemia into germ free mice, but not the microbiome from healthy mice, elicited the development of NAFLD in recipient mice [61].

NAFLD and NASH in humans tend to coincide with the existence of obesity and poor dietary habits, which makes it difficult to differentiate the effects of diet from those produced by the altered microbiome, and the metabolic changes that accompany both in liver disease. The abundance of some bacterial species in humans such as *Proteobacteria* [62], or *Bacteroides* [63] were associated with NAFLD, being higher in patients with NASH compared to healthy individuals. Consistent with this, the stool microbiome profile of children with NAFLD showed more abundant *Gammaproteobacteria* (phylum *Proteobacteria*) and *Prevotella* (*Bacteroides*) compared with the microbiota of obese children without NAFLD [64]. It is noteworthy that during NAFLD progression, there was an increase in *Proteobacteria* and decrease in *Firmicutes*, suggesting that the gut microbiome may not be stable during disease progression [65]. Short-term treatment with the non-absorbable antibiotic rifaximin in patients with steatosis and NASH led to an improvement in liver function, thus supporting the potential role of microbiome in the pathogenesis of the disease [66]. Similarly, long-term antibiotic treatment led to a decrease in small intestinal bacterial outgrowth, which was correlated with an improvement in liver function [67].

Taken together, these studies show that there may be correlations between intestinal bacterial composition and NAFLD or NASH. However, these observations are limited by the lack of reproducibility of the studies and the absence of a mechanism to explain their effects on NAFLD and NASH. Furthermore, in most studies, the microbiome is sampled from stool samples, being the bacterial composition different from the communities present in the most proximal areas of the intestine [68].

## 4. Genetic Factors

The interaction between the genetic status and the environmental factors may explain part of the inter-individual variability observed in the manifestation of the phenotype and severity of NAFLD. Unbiased genetic and epidemiological studies show strong evidence for the heritability of characteristic traits of NAFLD and have identified gene *loci* associated with the progression of the disease.

### 4.1. Heritability

Several clinical studies using family members demonstrate that first-degree relatives are at higher risk for NAFLD, suggesting a genetic predisposition to the disease [69,70]. Parental history is considered a risk factor for developing hepatic steatosis, even in metabolically healthy individuals [71]. As observed in twin studies, the genetic background determine susceptibility to NAFLD and the progression to fibrosis as well as the presence of risk factors for MS [72,73]. The heritability of hepatic steatosis, fibrosis, and serum alanine aminotransferase (ALT) was stronger in monozygotic than in dizygotic twins supporting the relevance of the genotype in the manifestation of NAFLD traits [74,75].

Epidemiological studies describe inter-individual differences in the NAFLD prevalence depending on the ethnicity. Hispanic Americans have the highest prevalence of NAFLD, followed by Americans of European descent, and African Americans having the lowest prevalence [76,77,78]. In this regard, African Americans show a different metabolic response to obesity and IR, as defined by reduced hypertriglyceridemia, steatosis, and accumulation of triglycerides in the visceral adipose tissue compared to Hispanics [79,80]. Asians have been associated with increased ballooning in liver histology studies [80] as well as increased hepatic steatosis and prevalence of IR [81] when compared to Caucasians, suggesting that this ethnicity may be at risk for NAFLD [82].

The first genome-wide association study (GWAS) performed in NAFLD showed that the contribution of the ancestry to the observed differences in the accumulation of hepatic fat content and susceptibility to NAFLD can be partly explained by the genetic status of the individuals [83]. Since then, numerous epigenetic and genetic studies including GWAS and candidate gene studies have defined our current understanding about the individual predisposition to NAFLD according to the gene architecture [84,85,86].

### 4.2. Gene Loci

Different variants of genes implicated in the cellular metabolism of lipids in the liver define the genetic risk factors for NAFLD. As such, the most relevant loci affecting NAFLD are PNPLA3, TM6SF2, GCKR, MBOAT7, and HSD17B13 (Table 1).

#### 4.2.1. Patatin-Like Phospholipase Domain-Containing Protein 3 (PNPLA3)

This gene encodes an enzyme that is expressed mainly in adipose tissue, retina, and the liver [87,88]. Within the liver, PNPLA3 is expressed in hepatocytes and hepatic stellate cells in the membrane of lipid droplets where it regulates the metabolism of lipids and retinol, respectively [129,130]. The expression of PNPLA3 is closely regulated by insulin at transcriptional and posttranslational dimensions, increasing the levels of this protein after feeding [131].

PNPLA3 has been widely studied in NAFLD since the missense variant rs738409 C>G encoding for the I148M allele of PNPLA3 was first discovered in 2008 by GWAS and reported to explain most of the genetic contribution to the hepatic triglyceride accumulation and tendency to NAFLD in patients of different ethnicity [83]. The PNPLA3 I148M variant has ever since been rigorously studied in liver disease [89,90,91,92,93]. In NAFLD, the I148M allele is associated with the severity of the disease and predicts mortality [132,133,134,135,136].

Functional in vitro studies to characterize the biological relevance of the PNPLA3 I148M variant in NAFLD showed that PNPLA3 I148M is a gene variation that causes loss of function [137,138]. However, in vivo data from studies using *knockout* (KO) mice revealed that lack of PNPLA3 protein did not affect the accumulation of fat in the liver, IR, or levels of liver enzymes [139]. Interestingly, mice with *knock in* and overexpression of the variant PNPLA3 I148M in the liver on high-fat diet reproduced the NAFLD phenotype observed in humans [140]. Additionally, PNPLA3 I148M protein was not efficiently ubiquitylated, avoiding proteasomal degradation [141]. For this reason, the accumulation of the mutant I148M protein on lipid droplets is the commonly accepted mechanism that drives the liver damage toward an accumulation of polyunsaturated fatty acids [142]. From a therapeutical point of view, efforts directed to lower the expression of PNPLA3 have been demonstrated to mitigate hepatic steatosis, liver inflammation, and fibrosis stage [143] in mice, while in humans, a gene variant associated with decreased expression of PNPLA3 reduced the effects of I148M on susceptibility to liver damage and steatosis [144].

#### 4.2.2. Transmembrane 6 Superfamily Member 2 (TM6SF2)

TM6F2 is a protein that is mainly expressed in the liver and small intestine where it regulates the intracellular trafficking and secretion of VLDL and cholesterol [94,95]. The precise molecular function of this protein requires more research, but in silico data suggest a catalytic activity as a sterol isomerase [145]. In the liver, this protein is expressed by hepatocytes, where it is localized in the endoplasmic reticulum and the Golgi apparatus.

The relevance of this locus in NAFLD was stablished in 2014 by an exome-wide association study that identified the missense variant rs58542926 C>T that encodes for the mutant TM6FS2 E167K [96]. In this study, the authors elegantly showed that carriers of this SNP had increased hepatic fat content and serum ALT, but reduced circulating levels of LDL-cholesterol and triglycerides regardless of the ethnicity. They clearly showed that the variant TM6FS2 E167K is associated with a loss of function as the protein expression levels in hepatocytes were lower in vitro. In the same direction, overexpression of TM6SF2 diminished hepatic cell steatosis [94]. The TM6SFS2 E167K variant is associated with an altered synthesis of VLDL-core lipids from polyunsaturated fatty acids [146] and failure in the secretion of large triglyceride-rich VLDL [147,148]. Defective production and secretion of VLDL and cholesterol from hepatocytes result in increased accumulation of fat in the liver and drives the already known liver damage and disease progression in NAFLD [149]. TM6SF2 E167K has been associated with a wide spectrum of liver disease [97,98,99,100]. However, unlike most NAFLD patients, those carrying the mutant version of TM6FS2 are protected from cardiovascular disease risk as they have diminished circulating levels of cholesterol and lipids because they are accumulated in the liver [150]. Interestingly, this variant has also been related to the development of T2DM, plausibly due to alterations in the aromatic amino acid metabolism [151].

#### 4.2.3. Glucokinase Regulator (GCKR)

This gene is expressed in the liver of vertebrates and encodes for a protein that acts as an allosteric inhibitor of glucokinase (GCK), an enzyme responsible for blood glucose homeostasis. GCK is activated by increased levels of glucose in the portal vein and catalyzes the beginning of the glycolytic pathway by phosphorylating the glucose that enters in the cell [101]. GCK has dual functions, stimulating the secretion of insulin in islet β-cells of the pancreas, and inducing the synthesis of glycogen by hepatocytes [152]. Hepatocytes help to regulate the blood levels of glucose by consuming it after a meal or producing and releasing endogenous glucose during a fasting period [153]. Therefore, a strict regulation of the activity GCK in the hepatocytes is required to guarantee a glucose-system homeostasis, which is the main function of GCKR. At the same time, GCKR activity is increased by fructose-6-phosphate (F6P) but suppressed by F1P.

The loss-of-function variant rs1260326 C>T SNP, which encodes for the P446L protein of GCKR, is related to steatosis in the liver and risk of NAFLD even in obese children and adolescents [102] and NASH-HCC [105]. A different variant of GCKR (rs780094 C>T, intron variant) was associated with high triglyceride levels, development of NAFLD, and severity of liver fibrosis, confirming the relevance of the GCKR gene status in the disease [103,104]. Cumulative gene variants in GCKR and other genes result in increased susceptibility and progression of NAFLD and metabolic syndrome [154,155].

Mechanistical studies using recombinant human GCKR wild type and P446L proteins showed that physiological levels of F6P failed to activate P446L GCKR, resulting in increased activity of GCK [156]. The consequence of this failure is a permanent activity of the glycolytic pathway and generation of malonyl coenzyme A (CoA), a precursor for fatty acids biosynthesis, which explains the accumulation of lipids in the liver and the increased risk for NAFLD.

#### 4.2.4. Membrane Bound O-Acetyltransferase Domain Containing 7 (MBOAT7)

MBOAT7 gene expression yields the enzyme lysophosphatidylinositol (LPI) acyltransferase, an endomembrane protein involved in the metabolism of lipids in the liver [106] that catalyzes the production of phosphatidylinositol (PI), a component of cell membranes.

A GWAS first identified the MBOAT7 variant rs641738 C>T as a risk locus in the pathogenesis of cirrhosis driven by alcohol [107]. Later studies associated this gene variant with susceptibility to develop NAFLD and severity of the disease in the entire spectrum [108,109]. Expression of MBOAT7 is reduced in both obese humans and rodents fed with a high-fat diet and MBOAT7 KO mice develop steatosis, hepatic inflammation, hepatocyte cell death, and increased expression of fibrosis-associated genes due to altered levels of PI and LPI in the liver [157]. Interestingly, a recent study described that accumulation of fat in the liver of individuals carrying a defective MBOAT7 can be explained by a fostered triglyceride synthesis due to an increased turnover of PI [158].

#### 4.2.5. 17-Beta Hydroxysteroid Dehydrogenase 13 (HSD17B13)

This gene encodes for retinol dehydrogenase, which is involved in steroid hormone signaling as well as bile acids and lipid metabolism [110]. HSD17B13 is expressed in the hepatocytes of the liver where it is localized in their lipid droplets [159].

Recently, it has been described that several polymorphisms including rs72613567, rs143404524, and rs62305723 have a protective role in liver injury as they have been associated with reduced serum hepatic ALT and AST levels, diminished risk for liver damage, progression toward HCC and liver-related mortality in alcoholic and nonalcoholic liver disease [111,112,113,114,115]. These SNPs introduce different gene variations-altered splicing, which result in truncated or stable HSD17B13 proteins with a marked loss of enzymatic activity. Unlike previous described loci, it seems that HSD17B13’s protective role in liver injury is associated with the retinol metabolism, inflammation, and fibrogenesis more than accumulation of lipids in the liver. These data suggest that efforts directed to reduce the expression of HSD17B13 may be beneficial in patients with liver disease. However, additional studies are needed to characterize the precise role of this gene.

## 5. Epigenetic Factors

Although the causal role of certain loci and NAFLD pathogenesis is undeniable, it is becoming clear that the rising prevalence of NAFLD cannot be explained exclusively by the contribution of environmental and genetic factors. Epigenetics, which constitutes the reversible and heritable change in gene expression without modification of the underlying nucleotide sequence, serves as a mechanistic bridge in this phenomenon (Table 1). As a matter of fact, there is a growing body of evidence portraying epigenetics as a crucial player in the pathogenesis and progression of NAFLD [160].

We can find examples of epigenetics on the basis of NAFLD as early as in embryo development. Maternal obesity, diabetes, or Western diet consumption [161] lead to an unfavorable intrauterine environment in which hepatic mitochondrial function in fetal liver is susceptible to damage. This exposure induces fetal metabolic reprogramming through epigenetic mechanisms, contributing to a lifetime risk of NAFLD, and likely to the severity and early onset of the disease in children [161]. For instance, as shown in mice [162], primates [163], and also in humans [164,165], parental environment, dietary habits, lifestyle, and behavior lead to an extensive alteration of the epigenome and chromatin structure of the offspring, which correlates with NAFLD development. Interestingly, there is evidence that these effects could be preventable by maternal exercising [166] or even bariatric surgery [167], indicating a significant potential for therapeutic intervention [168,169], a topic further reviewed in Sodum et al. [170].

### 5.1. DNA Methylation

The relationship between DNA methylation and other metabolic diseases has been extensively studied [171]. The amount of data available in NAFLD has also increased rapidly over the years [172]. NAFLD has mainly been associated with hypomethylation [173], most probably caused by an imbalance in methyl donor supply (reduced levels of folate) as has been suggested for T2DM [174]. Several studies have found, in support of this hypothesis, that a low methyl-donor diet can induce NAFLD in mice [175]. For instance, this phenomenon can be prevented in models of dietary-NAFLD induction by methyl-donor supplementation [176], which highlights the role of hypomethylation in hepatic steatosis. Additionally, it is known that several of these genes, whose transcription is dependent on DNA methylation in NAFLD, correlated with the severity of the disease. Genes involved in fibrogenesis such as TGF-β1, Collagen 1A1, and platelet-derived growth factor (PDGF) were hypomethylated and their expression was upregulated in more severe stages of NAFLD progression indicating risk of developing fibrosis [173,177].

Although the effect seen in NAFLD is hypomethylation, several interesting examples of hypermethylated genes with reduced expression can be found. Insulin-like growth factor-binding protein (IGFBP)-2 is often repressed in patients with NAFLD and NASH via methylation [116]. Interestingly, in models of dietary NAFLD induction, hypermethylation of IGFBP2 precedes the onset of hepatic steatosis development when mice were still metabolically stable, suggesting its potential as a risk indicator of liver disease development [178]. Reduction of IGFBP2 circulating levels have been observed in obese adults [179] and its hypermethylation state has been correlated with the risk of developing T2DM [180], suggesting that the dietary-dependent epigenetic status of IGFBP2 might play a role in the pathogenesis and interconnection between these diseases.

Another prominent example of a hypermethylated gene in NAFLD is Peroxisome Proliferator-Activated receptor gamma coactivator (PGC)-1α, a master regulator of various aspects of energy metabolism, especially fatty acid oxidation and mitochondrial biogenesis, which are features involved in the pathogenesis of fatty liver. Patients of NAFLD have decreased PGC1α expression due to promoter methylation, which correlates with mitochondrial defects and IR [117]. To be noted, fetal and neonatal PCG1α expression is compromised by maternal diets rich in fat and high BMI, respectively [166,181], indicating the importance of intrauterine environment and supporting fetal reprogramming hypothesis.

### 5.2. Hystone Methylation/Acetylation

Unlike DNA methylation, histone modifications are less understood in the context of fatty liver disease [182]. Nevertheless, some examples exist relating these epigenetic marks and NAFLD. For instance, the histone demethylase Jumonji domain containing (JMJD)-1C positively regulates the expression of genes involved in lipid metabolism. Deletion of JMJD1C in animal models protects from dietary-induced NAFLD and its overexpression increases liver fat content [183]. Similarly, the histone demethylase Plant homeodomain finger protein 2 (PHF2) induces hepatosteatosis when it is specifically overexpressed in the liver [184]. Among others, PHF2 regulates Carbohydrate-responsive element-binding protein (ChREBP) expression, a master regulator of lipid metabolism already associated with hepatic steatosis [185]. Interestingly, the histone acetyltransferase p300 is responsible for ChREBP acetylation. Given its role in the control of lipogenic genes, the overexpression of p300 activates ChREBP, which, in turn, leads to fatty liver and T2DM [186].

Sirtuin 1 (SIRT1), on the other hand, is a histone deacetylase that has been traditionally related to hepatic metabolic regulation [118]. As matter of fact, reduced levels of this protein have been found in both animal models and patients of NAFLD [119,120]. Similarly, the repression of SIRT1 in mouse liver is sufficient to induce hepatic steatosis [187], an effect that might be mediated by the key regulators of glycolysis and lipolysis, peroxisome proliferator-activated receptor (Ppar)-γ and Pparα as Sirt1 deletion impairs their function [188,189]. All together, these results highlight the importance of the epigenetic control of master regulators of metabolism such as ChREBP or Pparγ and the impairment of this regulation in NAFLD pathogenesis.

### 5.3. miRNAs

miRNAs are known to regulate multiple biological pathways involved in the pathogenesis of NAFLD such as lipid uptake, de novo lipogenesis, lipid oxidation, and hepatic lipid export, apoptosis, cell proliferation, or fibrosis [190]. In fact, at least a dozen of miRNAs have been strongly associated with NAFLD development, although the exact mechanism by which some of them contribute to the disease is still poorly understood [191]. As cell death increases with the progression of the steatohepatitis, several miRNAs are released either directly or packed in exosomes to the circulation, which also makes them potential biomarkers for the NAFLD status [125,192].

miR-33a and miR-33b, for example, negatively regulate the levels of ATP-binding cassette transporter 1 (ABCA1), which controls high-density lipoprotein biogenesis, thereby promoting high levels of circulating VLDL and triglycerides [193]. In the case of miR-34, it is known to be overexpressed in NAFLD patients where it targets SIRT1, a deacetylase involved in the regulation of energy homeostasis as mentioned [127,128]. Additionally, miR-34 can repress lipophagy by reducing mitochondrial oxidation, thus favoring the accumulation of lipids in the liver [194]. miR-122, on the other hand, is the most abundant miRNA in the liver, accounting for 70% of the miRNA pool [195]. Its reduction has been associated with steatohepatitis and the subsequent progression to fibrosis via increased lipogenesis and impaired lipid secretion in patients and animal models [121,124]. As a matter of fact, miR-122 regulates the expression of crucial genes in lipid metabolism such as Acetyl-CoA carboxylase 2 (ACC2), ChREBP, PPARγ, PPARα, or SREBP [121,122,123]. Additionally, and in contrast to liver levels, serum levels of miR-122 were higher in NAFLD patients than in the healthy controls, and further increased in NASH patients, suggesting not only an important role for this miRNA in NAFLD pathogenesis, but also its potential as a biomarker of liver damage [125,126].

## 6. Demographic Factors

### 6.1. Age

NAFLD and aging are known to be strongly correlated, with increasing age being one of the strongest epidemiological factors for NAFLD, NASH, and fibrosis [196,197,198,199,200]. Several studies have shown that the prevalence of NAFLD increases with age: 2.6% in children [201], 17.3% in adolescents [202], and up to 34% in adults [76]. Older age is not only a risk factor for the accumulation of fat in the liver, but it also increases the probability of mortality and disease progression to fibrosis and HCC in these elderly individuals. The relationship between age and fibrosis progression could be attributed to the longer duration of disease in the older age patients with NAFLD. However, most epidemiological studies have found a consistent relationship of NASH and fibrosis with increasing age, particularly after the fifth decade of life.

### 6.2. Gender

Unlike what happens with age, the influence of sex on the prevalence and course of NAFLD is not so clear. Available studies are divided between both sex, some have shown a female preponderance, while others have shown a male preponderance in the prevalence of NAFLD [203]. In an interesting study published in 2011 [204], women had a significantly higher prevalence of NAFLD compared with men, but males had a more adverse NAFLD phenotype, related to a higher prevalence of the MS, thicker visceral adiposity, higher fasting glucose, transaminase levels, and systolic blood pressure. However, they had lower levels of serum adiponectin and high-density lipoprotein than females [204]. The higher prevalence in women in this study is in contrast to other reports that have found a higher prevalence of NAFLD in men [20,78].

## 7. Environmental Risk Factors

In recent years, NAFLD has become a biological marker of social affluence and sedentary lifestyle [2,24]. As we have already seen, many metabolic risk factors play a very vital role in the development of NAFLD. However, there is growing evidence to support the potential effects of exposure to some environmental factors on the development of liver disease. Environmental exposure to toxins related to landfills has been associated with an increased prevalence of autoimmune liver disease [205,206]. For this reason, more and more attention is being paid to the effects of environmental factors on liver diseases including NAFLD.

### 7.1. Diet and Lifestyle

Numerous studies have suggested that dietary composition may predispose people to NAFLD. In developed societies, human nutrition has undergone drastic changes in recent decades that, combined with a decrease in the population’s physical activity, has triggered a marked imbalance between energy consumption and energy expenditure [7]. The rise in knowledge-based jobs has also contributed to the increasing prevalence of obesity. Economic globalization has led to changes in dietary habits such as increased consumption of carbohydrates, animal proteins, refined sugars, and additives, which play an important role in the development of NAFLD [207,208,209]. This energy imbalance can lead to a huge increase in obesity and IR, which itself is also a contributing factor for NAFLD [207], as mentioned.

Leslie T. et al. reported in 2014 [210] that NAFLD patients tended to reside in areas with many options of food source including grocery stores, restaurants, and fast-food places. Additionally, people with NAFLD were more likely to have the least healthy eating habits and reported eating more frequently at restaurants [210]. Other studies in patients with NAFLD have documented increased consumption of low-nutrient, high-sodium, and high -fat foods, especially high-fat diets derived from meat and lower amounts of fresh fruits [211,212]. In addition, people with fatty liver were found to have lower levels of physical activity and longer sitting time compared with healthy individuals [213,214,215].

One of the underlying mechanisms that could lead to the onset NAFLD from this particular scenario might be epigenetics, as mentioned before; the mechanism that could also be responsible for the IR that this population suffer [216,217]. Interestingly, if this is the case, non-pharmacological approaches such as dietary intervention [168] and physical exercise [218] could be used in order to tackle the increasing prevalence of these diseases. So far, there has been very promising results regarding the effect of exercise in the overall NAFLD status [219,220], directly or mediated by the improvement in comorbidities like IR [221]. Additionally, easy-to-implement dietary modifications such as caloric restriction [222], intermittent fasting [223], and ketogenic diet [224] have also reported improvement and even protection from the progression of NAFLD. These data together suggest that changes in lifestyle based on dieting and exercise, which, in turn, might be restoring the epigenetic landscape altered in NAFLD, is the most promising strategy to fight the disease so far.

### 7.2. Smoking

Several recent studies have reported the relationship between smoking and the incidence of an accelerated disease progression and advanced fibrosis in NAFLD [225,226,227,228,229]. Yuan et al. [230] demonstrated in 2009 that exposure to tobacco smoke could accelerate the development of experimental NAFLD, and enhances steatogenesis caused by a high-fat diet. Furthermore, in this study [230], the authors indicated that tobacco smoke could alter the regulatory effect of AMP kinase on lipid metabolism. The study builds on a previous report, showing that chronic exposure to environmental smoke in apoB transgenic mice is associated with atherosclerotic plaque initiation features [231]. Increased hepatic lipogenesis has been shown to account for about 30% of triglyceride accumulation in steatotic livers [232], thus investigators have subsequently reviewed the impact of second-hand smoke on lipogenic pathways in the liver.

Zein et al. [233] demonstrated that smoking can accelerate the progression of human NALFD, supporting the recommendation to quit smoking in patients with NAFLD. This recommendation is in addition to general recommendations for an appropriate dietary or lifestyle approach for NAFLD patients [234].

### 7.3. Air Pollution

According to some epidemiological studies, exposure to environmental particles is positively associated with increased morbidity and daily mortality caused by diseases closely related to life habits including ischemic heart disease [235] or T2DM [236]. Studies on the harmful effects of air pollutants on atherosclerotic cardiovascular diseases [228] have shown that they could be mediated through oxidative stress and IR [237], factors also known to play a very important role in the pathogenesis of fatty liver [238]. Therefore, it could be assumed that exposure to such environmental factors might also be associated with NAFLD.

Long-term exposure to PM_2.5_ can affect NAFLD through different ways [239]. In 2007, Folkmann et al. [240] published an interesting study in which they demonstrated a direct effect of diesel exhaust particles in the liver of dyslipidemic mice, causing inflammation and oxidative damage to DNA. Tan et al. [241] demonstrated shortly thereafter that environmental particles reaching the liver had the potential to induce cytokine secretion by Kuppfer cells, which can trigger inflammation and collagen synthesis by hepatic stellate cell, suggesting that the exposure to environmental particles < 2.5 µm (PM_2.5_) may be an important risk factor for NAFLD progression. PM_2.5_ may also prompt the expression of proinflammatory factors in adipocytes [242,243,244], activating Kuppfer cells [245] and causing inflammation in NASH [246].

In 2009, Sun et al. [242] found that mice inhaling PM_2.5_ for more than 30 min showed an abnormal liver insulin signal, with an altered expression of endothelial NOS and protein kinase C. More recently, Yin et al. [247] demonstrated that PM_2.5_ exposure could inhibit the expression of PPARα and PPARγ, inducing hepatic steatosis, inflammation, and IR. According to numerous studies, oxidative stress is the main cause of damage of PM_2.5_ in cells, either through direct effects on structure and function of biological macromolecules like DNA, lipids, and proteins, or indirectly through the activation of intracellular oxidant pathways [248,249,250,251].

Despite the results on the inflammatory and oxidative properties of air pollutants, the lack of data supports the need of more studies on the effects of environmental factors, notably air pollution, on NAFLD.

## 8. Summary

NAFLD is a growing health problem worldwide. Recently, an international expert group has agreed to change the name of NAFLD to metabolic (dysfunction) associated fatty liver disease (MAFLD) [252,253]. Overall, all studies confirm that NAFLD is a complex disease that is affected by metabolic and environmental factors, along with genetic and epigenetic predispositions, involving multiple organs and diverse mechanisms. The exact contribution of each factor in the development of NAFLD is unknown and may vary by geographic location. Therefore, future studies are needed to better understand the pathogenic mechanisms developed in NAFLD with the aim to personalizing the treatment of patients and being able to improve their outcomes.

## Figures and Tables

**Table 1:** Genes associated with NAFLD pathogenesis and progression.

Gene	Tissue Expression	Function	Main Alterations/Variants	Effect
*PNPLA3*	Liver, adipose tissue and retina	Lipid droplet remodeling. Lipid metabolism [87,88]	Loss of function mutations: rs738409 C>G/p.I148M [83]	↑ NAFLD, NASH, fibrosis, HCC [89,90,91,92,93]
*TM6SF2*	Liver and small intestine	VLDL and cholesterol trafficking and secretion [94,95]	Loss of function mutations: rs58542926 C>T/p.E167K [96]	↑ NAFLD, NASH, fibrosis [97,98,99,100]
*GCKR*	Mainly liver	Regulation *de novo* lipogenesis. Blood glucose homeostasis [101]	Loss of function mutations: rs1260326 C>T/p.P446L and rs780094 C>T/intronic [102]	↑ NAFLD, NASH, fibrosis [102,103,104,105]
*MBOAT7*	Ubiquitous, Liver enriched	Phosphatidylinositol remodeling [106]	Loss of function mutations: rs641738 (C>T)/? [107]	↑ NAFLD, NASH, fibrosis [108,109]
*HSD17B13*	Ubiquitous, Liver enriched	Lipid droplet remodeling. Retinol metabolism [110]	Loss of function mutations: rs72613567 A>T/intronic and rs143404524/frame shift [111,112,113,114,115]	Protective effect. ↓ NAFLD, NASH, fibrosis, HCC [111,112,113,114,115]
*IGFBP2*	Mainly liver and kidney	IGF factors transportation [116]	Hypermethylation. Reduced expression. [116]	↑ NAFLD [116]
*PGC1α*	Muscles, liver, adipose tissue and kidney	Energy metabolism and mitochondrial biogenesis [117]	Hypermethylation. Histone hypoacetylation. Reduced expression [117]	↑ NAFLD, NASH [117]
*SIRT* *1*	Ubiquitous	Histone deacetylase. Regulates several genes involved in metabolism control [118]	Reduced expression [119]	↑ NAFLD [119,120]
*miR-122*	Ubiquitous, Liver enriched	Regulation of lipid metabolism and fibrogenesis [121,122,123]	Reduced expression in the liver [124]	↑ NAFLD, NASH, fibrosis, HCC [125,126]
*miR-34*	Ubiquitous	Regulates lipophagy. Negatively regulates *SIRT1* [127,128]	Liver overexpression [127,128]	↑ NAFLD [127,128]

We presented only genetic variants and epigenetic-related factors significantly associated with predisposition toward development and/or progression of NAFLD described in the manuscript. Other genetic and epigenetic factors generally predisposing to insulin resistance or dysmetabolism without clear and funded relation to NAFLD are not reported.

## Data Availability

Not applicable.
